# Measuring US fertility using administrative data from the Census Bureau

**DOI:** 10.4054/demres.2022.47.2

**Published:** 2022-07-07

**Authors:** Katie Genadek, Joshua Sanders, Amanda Stevenson

**Affiliations:** 1 United States Census Bureau, USA.; 2 University of Colorado Boulder, USA.

## Abstract

**BACKGROUND:**

Longitudinal data available for studying fertility in the United States are not representative at the state level, limiting analyses of subnational variation in US fertility. The US Census Bureau makes available restricted data that may be used for measuring fertility, but the data have not previously been described for a scholarly audience or used for fertility research.

**OBJECTIVE:**

This paper describes and analyzes restricted-use administrative birth data available through the Census Numident for nearly all US births for more than the last century. Within these data, most births since 1997 are linked to parents through the Census Household Composition Key (CHCK). These analyses are designed to illustrate the scope and limitations of these data for the study of US fertility.

**METHODS:**

We describe the creation and content of the Census Numindent and CHCK data sets and compare the data to published US vital statistics. We also analyze the geographic coverage of both data sets and compare the demographic composition of the new data sources to national demographic composition. We further illustrate how these novel data sources may be used by comparing them to survey responses at the individual level.

**CONTRIBUTION:**

This paper describes an underutilized source of national US data for studying fertility, shows the quality of these data by performing analyses, and explains how scholars can access these data for research.

## 1. Introduction

Since the family-building model came to prominance in the 1970s (Menken 1974; Trussell and Menken 1978), demographers have agreed that individuals’ prior fertility is key to understanding their reproductive lives, but longitudinal data on individuals’ childbearing in the United States is only representative at the national level. This is consequential, as efforts to examine fertility in the context of fertility delay and decline are increasingly focused on parity, or the number of births women have had (Hartnett and Gemmill 2020; Beaujouan and Berghammer 2019; Zeman et al. 2018). As state-level policies and conditions continue to hold substantial demographic salience, the absence of data facilitating comparisons across subnational geographies limits demographic research (Riley et al. 2021; Montez et al. 2020; Chetty et al. 2014). In this paper we describe data sources which might be used to fill this gap in US data on parents and children. The data we describe may be anonymously linked at the individual level using a Census Bureau-assigned key that links to most Census Bureau administered surveys, increasing their utility for demographic research.

Restricted census, survey, and administrative data are available from the Census Bureau through the Federal Statistical Research Data Centers (FSRDCs) to researchers on approved projects. The data holdings change over time and these changes can be particularly substantial among data derived from administrative records. Administrative data in general often lack comprehensive documentation because their primary purpose is not academic research. Data of this type held by the Census Bureau are no exception, and the absence of documentation can present a barrier to researchers’ knowledge of and ability to use the Census Bureau’s substantial data holdings. This paper describes the Census Numerical Identification (Numident) and the Census Household Composition Key (CHCK) data files. The Census Numident and CHCK files are derived from the Social Security Administration (SSA) Numident, and they provide birth information and links between children and birth parents as reported on Social Security Number (SSN) applications. In addition, we analyze these data files to assess their quality and comparability to vital statistics data and survey data. We present results from these analyses so researchers understand the available data. We conclude by discussing the use of these restricted data for fertility research.

## 2. Data description

### 2.1 SSA Numident

The SSA uses the Numident to maintain records of Social Security Number (SSN) holders in the United States. While SSNs were created and issued starting in 1936, electronic tracking of SSN information in the SSA Numident began in 1972. All existing SSN information has been digitized and is included in the electronic SSA Numident file (Puckett 2009). The SSA Numident contains all recorded interactions individuals have with the SSA related to SSNs. Thus, it includes information on SSN applications, claim records, death reporting, and requested changes to SSN information. There are now more than one billion transactions within the SSA Numident for approximately 518 million SSN holders in the Numident (Finlay and Genadek 2021).

Prior to 1989, individuals or individuals’ parents filled out the SSA application for a Social Security Card, Form SS-5, which included date of birth, place of birth, gender, race, citizenship status, parents’ names, and parents’ SSNs. Starting in 1989, the SSA entered into agreements with each state in order to enumerate individuals at birth. When infants are now born in hospitals and birthing centers, the parents are asked if they would like the birth certificate data to be transmitted to the SSA to create an SSN for the individual at birth. SSA publications suggest that more than 95% of births in the United States are assigned an SSN through this enumeration at birth (Puckett 2009). That information is given to the state’s vital statistics office, and the vital statistics office sends the information from the birth certificate to the SSA to create a record for the infant and issue an SSN. Selected information from the birth certificate, including name, date of birth, place of birth, mother’s name, mother’s SSN, father’s name, and father’s SSN, are shared with the SSA. If parents do not elect to have their child enumerated at birth by the SSA, they can apply for an SSN through an SSA application office. Moreover, adoptive parents can apply for new SSNs for adopted children through the SSA prior to or following adoption, which include their adoptive parents’ information rather than the birth parents’ information.^3^

The Census Bureau obtains the SSA Numident data in quarterly updates from the SSA for the purposes of improving Census Bureau survey and decennial census data, performing record linkage, and using the data for research and statistical projects. While most information from the SSA Numident is included in this transfer, the Census Bureau does not receive the parents’ SSN information from an individual’s SSN application, although they do receive parents’ names. The Census Bureau creates two research files useful for measuring fertility by capturing birth information using the SSA Numident file. The first is the Census Numident file and the second is the CHCK.

### 2.2 Census Numident

The Census Bureau creates the Census Numident by processing quarterly updates from the SSA transaction-level data to create a person-level research file that includes the history of individual-level interactions with the SSA Numident. Like the SSA Numident, the Census Numident is a cumulative file. In the Census Numident the SSN is replaced with a Census Bureau Protected Identification Key (PIK), a unique anonymous identifier. Some other Personally Identifying Information (PII), including name, is removed from the Census Numident file. The resulting data file, with the PIK, is then made available to Census Bureau staff and external researchers for approved Census Bureau production and research projects.

The Census Numident includes one record per person who has received an SSN in the United States. The scope of information in an individual’s Census Numident record varies based on when the individual received an SSN, how the individual applied for an SSN, and if the individual has interacted with the SSA, such as for a name change. In general, most records include complete date of birth, place of birth, and sex. The universe for this file is all individuals receiving an SSN, so unlike the birth records from birth certificates in the United States, it includes people born outside of the United States who apply for an SSN. However, place of birth is obtained for all SSN applicants.

### 2.3 Census Household Composition Key (CHCK)

In addition to the Census Numident, the Census Bureau creates the CHCK files. These files are crosswalks of individuals aged 0–19 with a PIK linked to their mother’s and father’s PIKs. The file also includes the child’s exact birth date as reported to the SSA. This is not the same file as the SSA KIDLINK database (or Internal Revenue Service (IRS) research file DM-2) which uses parents’ SSNs on the child’s SSN application to directly link parents and children.^4^ Without the SSNs of parents, the Census Bureau assigns PIKs to the parents in the child’s Numident record using the Person Identification Validation System (PVS), which probabilistically assigns PIKs to respondents in surveys generally by matching information in the survey to a composite reference file with PIKs (Wagner and Layne 2014). In this case, PVS is used to assign PIKs to the parents of the children in the Census Numident based on the parents’ reported names (Luque and Wagner 2015). In addition to using the names, the child and parent pair in the Census Numident must be confirmed at the same address within the PVS reference file or the decennial census. This coresidential requirement is necessary to limit and refine the linkages based on names alone. The PVS reference file addresses are extracted from trusted federal administrative records, which have been previously processed through the PVS system at the Census Bureau. Detailed information on the creation of the CHCK file is documented in Luque and Wagner (2015), which describes the creation of a preliminary version of the CHCK file (at the time called Census Kidlink) using the 2007 Census Numident.

The CHCK file is not cumulative. Instead, yearly versions of the CHCK are created based on vintages of the Census Numident. For each vintage year of the Numident, the corresponding CHCK file includes parent links for observations aged 0–19. The first CHCK file is available for Census Numident vintage 2016, and thus the births start in 1997. The CHCK file for 2019 includes birth counts complete through 2018.^5^

## 3. Quality assessment and analyses

### 3.1 Birth counts compared to vital statistics

To assess the quality and completeness of the birth records obtained through the Census Numident we compare the United States-born individuals in the Census Numident to the births occurring in the United States published by the Center for Disease Control and Prevention’s (CDC) National Vital Statistics System (NVSS).^6^ Table 1 shows yearly birth counts starting in 1910 based on birthdates for all individuals in the Census Numident (Column 1) and yearly counts for those born within the United States (Column 2).^7^ In the most recent years, nearly all births recorded in the Census Numident occur in the United States. Also included in Table 1 is the total count of yearly births occurring in the United States obtained from the CDC’s NVSS.^8^ The number of yearly births in the Census Numident is very close in number to the reports from the NVSS, which is especially expected starting in 1989 because of the enumeration at birth being closely tied to birth certificates. However, even prior to 1989, the Numident captures just slightly more births than published through the NVSS back to 1970. This is shown clearly in column 4, which shows the proportion of US births in the Census Numident compared to the NVSS. The slight difference, with more births found in the Census Numident than the vital statistics, is potentially the result of a number of factors, including inaccurate place of birth information reported to SSA, and some US births outside of hospitals without birth certificates being excluded from the vital statistics counts. Prior to birth year 1969, the Numident generally contains fewer births than reported by vital statistics, though it is near or above 0.90 prior to 1920.^9^

At the national level, the birth data in the Census Numident look complete and comparable to the birth reports from the NVSS. To further understand the coverage of the Numident birth data, we count births by state of occurrence between 2009 and 2018 using the place of birth information in the Census Numident and compare them to the published births by state of occurrence from the CDC NVSS.^10^ Table 2 shows the state-level coverage of the Census Numident birth information and includes counts of births for all US territories combined.^11^ There is minimal variation in state-level coverage of births by the Census Numident, with the proportion of births in the Census Numident divided by the CDC NVSS ranging from .994 in Wisconsin to 1.052 in Maryland, with 26 states being between 0.999 and 1.001. While we present results for state-level births, detailed place of birth is also included in the Census Numident.

### 3.2 Analyses of children linked to parents

We combine four CHCK files by starting with the 2016 version and adding any additional births that appear in each successive file through the 2019 version. We keep one child–mother link and/or one child–father link if a child is linked to different mothers and fathers across years.^12^ Table 3 shows the birth counts and parental linkages for each birth occurring in at least one of the CHCK files, covering birth cohorts of 1997–2019. Parental linkages improve as time progresses over the first few years after a birth because the parent–child pair must be confirmed at an address in the PVS reference file or the decennial census, a requirement which is difficult to meet immediately after a birth because there is often a delay in the infant appearing in the administrative records. As shown in Table 3, only 80% of the births in 2018 and about 88% of births in 2017 are linked to any parent. Thus, the linkage rates of future CHCK versions will increase for children born in 2017 and 2018, though linkage rates in the most recent birth years will always be slightly lower than earlier years. In all of the birth cohorts prior to 2017, an average of 94.5% of all births are linked to at least one parent. The parental linkage rates for the birth cohorts of 1997–2016 are slightly higher for those born within the United States, 95.6%.

Table 3 also shows the percentage of children in the CHCK linked to a mother, linked to a father, or linked to both, by birth year. In most years, about 15% of children are linked to only a mother, while about 2.5% are linked to just a father, and the remaining 82.5% are linked to two parents.^13^ These parent linkages are based on the names on the SSN application and documented coresidence with a parent. While some of the two-parent linkages are missing due to issues with the probabilistic name matching and coresidence with a parent, SSN applications do not always include information for both parents, as fathers’ names are often not included on birth certificates.^14^

The children missing links to their parents in the CHCK file are not expected to be random. The linkage of children to parents in the CHCK file is first limited to parents that have been assigned a PIK. If a child is born in the United States to a parent that has not been assigned a PIK (they do not have an SSN or an Individual Taxpayer Identification Number (ITIN)), it will not be possible to link them together. Linkages will also not be made when the parents’ names in the SSN application are inaccurate or the probabilistically matched parent–child pair could not be confirmed at a location in the PVS reference file. Finally, the children may not be coresiding with the parent whose name is listed on the birth certificate or given to the SSA. Thus, we anticipate biases in the CHCK data when compared to the overall national population. Table 4 shows basic demographic characteristics (sex, race/ethnicity, birthplace) for those born between 1997 and 2018 linked to at least one parent in the CHCK, in the full Census Numident, and in the weighted 2019 1-year American Community Survey (ACS) Public Use Microdata (PUMS) (Ruggles et al. 2021). The weighted ACS PUMS is nationally representative, and thus provides the national comparison.

The three data sets have similar proportions of men and women, but the race/ethnicity breakdown is slightly different. For those with parent links in the CHCK, 54.61% are White non-Hispanic, while 22.69% are Hispanic. The full Census Numident is similar, with 53.65% of the respondents being White non-Hispanic and 23.89% being Hispanic; however, when we look at the weighted 2019 ACS, which is the nationally representative estimate, 50.79% of these birth cohorts are White non-Hispanic and 24.84% are Hispanic. There are smaller yet similar differences in most of the other non-White groups (Asian, Black, and Other), where the ACS has a larger percentage of the weighted total than the CHCK or the Numident.

**Figure 1:**
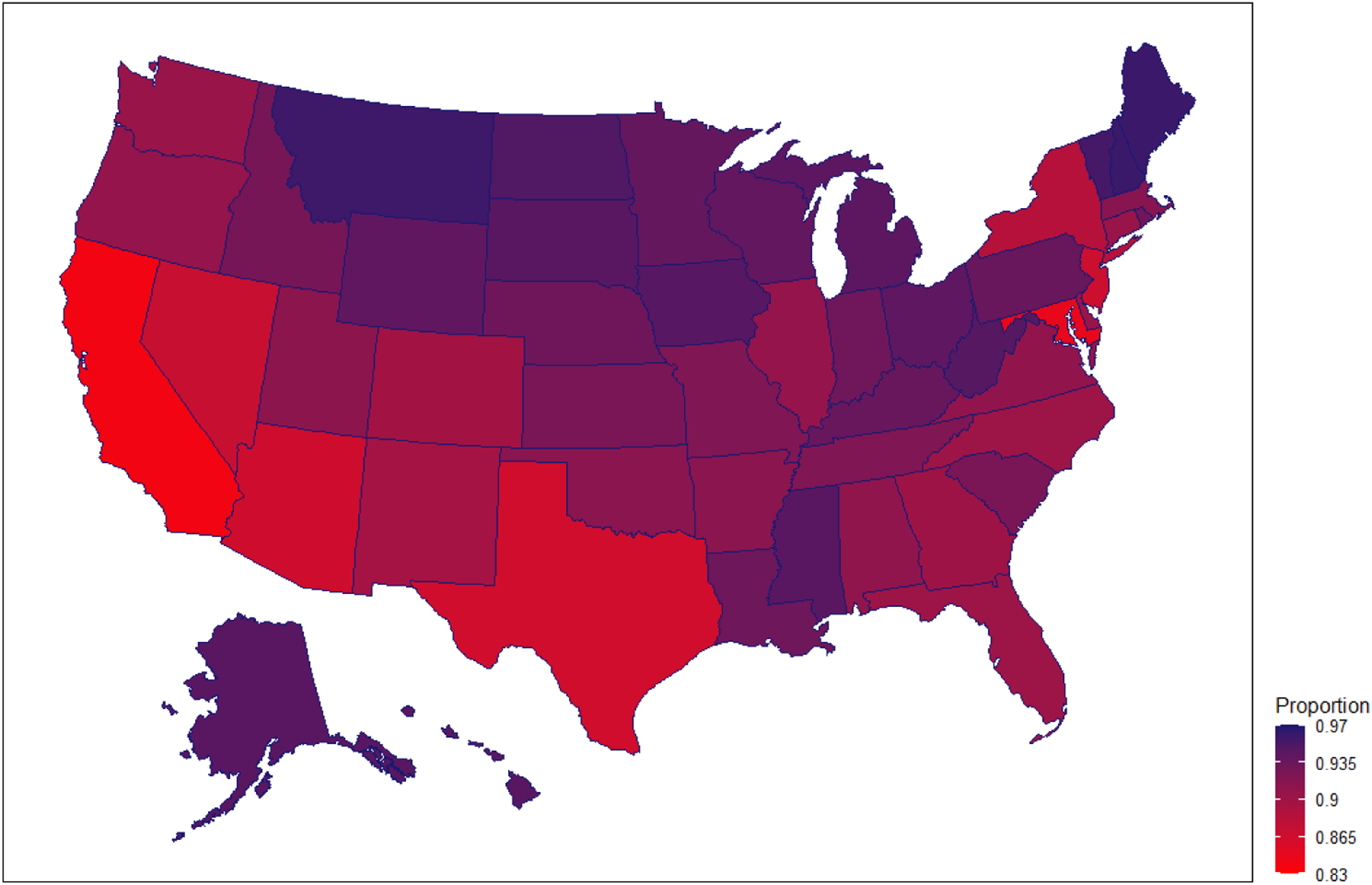
Proportion of births linked to a parent in the in Census Household Composition Key (CHCK), 1997–2018 *Notes*: Calculations from the 2016–2019 CHICK files. All results were approved for release by the US Census Bureau, authorization number CBDRB-FY21-ERD002-016.

In addition to demographic variation in the linkage of children to parents in the CHCK, there is also geographic variation. Figure 1 shows a map of the United States with state-level parent–child linkage rates from the CHCK data. The darkest areas on the map are states where the proportion of births linked to parents is between .935–.97, while the lightest states are between .83–.865. Similar to PIK rates in general (Rastogi et al. 2012), states in the southwest have the lowest linkages between children and parents. This is likely due to fewer parents in these states having SSNs and ITINs than in other states.

### 3.3 Comparing administrative fertility data to survey data

The Census Numident provides an administrative record of births based on birth certificates since 1989, and the CHCK files include probabilistic links between children and parents listed on birth certificates since 1997. This rich data source on births is a near complete record of all births occurring in the United States, and parent links are made to around 90% of the births since 1997. In order to further analyze the birth and parent links contained in the CHCK file, we linked all respondents born after 1997 and under age 19 at the time of the 2005 through 2019 1-year ACS surveys to the CHCK file using the Census Bureau-assigned PIK.

Table 5 shows the total number of children meeting the age and birth year criteria by year of the ACS. Of those in the universe, it also shows the total number and percentage that were assigned a PIK. Approximately 85%–92% of the children in the ACS were assigned a PIK and were thus eligible to be linked to the CHCK. Panel A of Table 5 contains estimates of children linked to mothers. Column 4 shows the total number of children that were linked to a mother in the CHCK. Nearly 95% of children with a PIK had a mother indicated in the CHCK. Column 6 shows the number of these children that reside in the ACS household with the mother, as indicated in the CHCK. Approximately 80%–85% of children with PIKs in the ACS reside with the mother assigned to them in the CHCK. When we look at those linked to a mother in the CHCK, about 85%–90% are living with the mother indicated in the CHCK at the time of the ACS. While this suggests there may be error in the assignment of mothers to children in the CHCK, this result also shows the universe of the CHCK file, in which the mother’s information is coming from the SSA Numident via a birth certificate and children may not always reside with that mother.^15^ Panel B of Table 5 shows the same estimates for fathers. A smaller percentage of children in the CHCK are linked to a father than to a mother, and the percentage of linked children who are residing with the father indicated in the CHCK is about 10% less than for mothers. This is expected, as many children are born to mothers without a father present, and children are more likely to reside with the mother if the parents do not live together (Smock and Schwartz 2020).

The CHCK file is organized at the child level, yet it is possible to use the data with the parents as the primary unit of analysis. Specifically, the data can be reshaped to focus on mothers, with their children and children’s birthdates from the CHCK indicating births to the woman. Using the data in this way allows for the study of birth parity. We focus on mothers with births in the previous year in the CHCK and then link these mothers to the ACS. The ACS survey asks women between the ages of 15 and 50 if they gave birth in the past twelve months. In addition to using the ACS to look at children linked to their parents, we analyze women of reproductive age in the ACS and their response to this fertility question, the children residing in their household, and their link to children in the CHCK.

Table 6 presents the results of these comparisons. Column 1 shows the total number of ACS respondents in the universe for the fertility question, or women of reproductive age (ages 15–50) in each year of the ACS since 2005. The number of those indicating they gave birth in the last year is shown in Column 2, and is generally around 5%, as shown in Column 3. About 74%–81% of the women indicating they gave birth in the last year in the fertility questions also had a child under age 1 living with them in their ACS household at the time of the survey (these percentages are shown in Column 4). Although not shown in Table 6, an additional 7.5%–9.0% of women indicating they gave birth in the previous year in the ACS have a child of age 1 living with them but do not have a child under age 1 living with them. Thus, close to 90% of the women who gave birth in the last year are living with a baby at the time of the ACS, and the other 10% of women are either not living with the infant they birthed in the past year or there is misreporting in their fertility status or the age of their children.^16^

The next panel of Table 6 shows similar information, but these columns present the percentage of reproductive-age women in the ACS that gave birth in the past year according to the CHCK, rather than the ACS fertility question. The percentage of women with a CHCK birth in the last year (Column 6) is slightly lower than the ACS fertility question, ranging between 3.55% and 4.28%, though a larger percentage of these women are residing in the household with the CHCK-linked child at the time of the ACS than women indicating they gave birth in the last year in the survey question (Column 7).

In the third panel of Table 6 we limit the sample to women who indicated they had a birth in the last year in the ACS and had a birth according to the CHCK. We find that between 3.23%–3.86% of reproductive-age women in the ACS in a given sample year gave birth according to both data sources. As shown in the final column, between 63.41%–71.55% of those that reported a birth in the ACS also gave birth based on linkages in the CHCK.

Comparing the CHCK birth information to that in the ACS provides insight into what the data captures. The limitation of many administrative records is the inability to measure US residents who do not have SSNs and ITINs, and the issue is present in these data. There are residents in the United States captured by surveys like the ACS that are not captured in our administrative records. However, for those captured by the Census Numident and CHCK, our ability to observe most of the linked children and parents residing together in the survey data demonstrates that the assignment of children to parents is of high quality.

## 4. Using Census Bureau data for fertility research

We have shown that the counts of births in the restricted-use Census Numident are similar to those from vital statistics. While the Census Numident includes all births assigned SSNs in the United States and the vital statistics include all births occurring in the United States, when we limit the Census Numident to births occurring in the United States the counts are very similar, even at the state level. The CHCK data, which provides linkages between children and their parents at the time of birth from 1997 onward, make the birth records in the Census Numident more useful for research. We find that over 90% of births are linked to at least one parent.

The Census Numident and CHCK data are an excellent resource for research on US fertility. These restricted-use data are available through the Census Bureau and the FSRDC network, providing an opportunity for detailed analyses of fertility and the family in the United States. The FSRDC network currently includes 31 physical research centers at universities and research institutions, and many researchers are now accessing the data through the network virtually.^17^ All research is performed within the restricted environment, and all results are reviewed before release to ensure the confidentiality of respondents. Researchers from any institution can apply to use the Census Numident and CHCK data through the standard Census Bureau FSRDC application procedures, starting by reviewing the research proposal process documentation and by contacting the closest physical FSRDC.^18^

Within the Census Bureau’s Data Linkage Infrastuture, the research possibilities grow when Census Numident and CHCK data are linked at the individual level to other data held at the Census Bureau. Administrative and survey data with detailed household location information, combined with the detailed place of birth information in the Census Numident, allow for substate analyses of births not possible with most fertility data. It is also possible for researchers to measure parity and estimate fertility by parents’ characteristics for nearly all births occurring in the United States using the CHCK linked to survey and administrative data. We link the CHCK data with ACS microdata, finding that substantial numbers of linked parent–child pairs are living together shortly after the child’s birth. While these rich data present robust opportunities for research, our linkage between CHCK and the ACS illustrates – in a small way – how using linked administrative and survey data can generate analytic challenges, since not all women who reported giving birth in the previous year in the ACS were assigned a birth in the previous year in the CHCK. However, with careful research design, these data can provide a new source of longitudinal, nearly full-count data on fertility in the United States.

## Figures and Tables

**Table 1:** Yearly births in the Census Numident and the CDC NVSS

Birth year	(1)	(2)	(3)	(4)
	Births in Numident	Births in USA in Numident	Births in USA from CDC NVSS	Numident USA Births/CDC NVSS
1910	2,566,000	2,425,000	2,777,000	0.873
1911	2,524,000	2,382,000	2,809,000	0.848
1912	2,668,000	2,484,000	2,840,000	0.875
1913	2,684,000	2,495,000	2,869,000	0.870
1914	2,807,000	2,593,000	2,966,000	0.874
1915	2,780,000	2,559,000	2,965,000	0.863
1916	2,789,000	2,570,000	2,964,000	0.867
1917	2,823,000	2,598,000	2,944,000	0.882
1918	2,979,000	2,726,000	2,948,000	0.925
1919	2,917,000	2,644,000	2,740,000	0.965
1920	3,128,000	2,786,000	2,950,000	0.944
1921	3,171,000	2,837,000	3,055,000	0.929
1922	3,104,000	2,738,000	2,882,000	0.950
1923	3,093,000	2,721,000	2,910,000	0.935
1924	3,152,000	2,780,000	2,979,000	0.933
1925	3,092,000	2,707,000	2,909,000	0.931
1926	3,030,000	2,634,000	2,839,000	0.928
1927	3,039,000	2,639,000	2,802,000	0.942
1928	2,977,000	2,553,000	2,674,000	0.955
1929	2,877,000	2,457,000	2,582,000	0.952
1930	2,941,000	2,475,000	2,618,000	0.945
1931	2,771,000	2,331,000	2,506,000	0.930
1932	2,798,000	2,323,000	2,440,000	0.952
1933	2,670,000	2,194,000	2,307,000	0.951
1934	2,766,000	2,263,000	2,396,000	0.944
1935	2,802,000	2,265,000	2,377,000	0.953
1936	2,795,000	2,240,000	2,555,000	0.877
1937	2,856,000	2,288,000	2,413,000	0.948
1938	2,965,000	2,367,000	2,496,000	0.948
1939	2,965,000	2,349,000	2,466,000	0.953
1940	3,097,000	2,445,000	2,559,000	0.955
1941	3,209,000	2,577,000	2,703,000	0.953
1942	3,560,000	2,877,000	2,989,000	0.963
1943	3,657,000	2,964,000	3,104,000	0.955
1944	3,555,000	2,823,000	2,939,000	0.961
1945	3,547,000	2,778,000	2,858,000	0.972
1946	4,171,000	3,329,000	3,411,000	0.976
1947	4,643,000	3,746,000	3,817,000	0.981
1948	4,497,000	3,587,000	3,637,000	0.986
1949	4,533,000	3,610,000	3,649,000	0.989
1950	4,576,000	3,620,000	3,632,000	0.997
1951	4,724,000	3,793,000	3,820,000	0.993
1952	4,893,000	3,903,000	3,909,000	0.998
1953	4,956,000	3,954,000	3,959,000	0.999
1954	5,134,000	4,078,000	4,071,000	1.002
1955	5,198,000	4,109,000	4,097,000	1.003
1956	5,326,000	4,216,000	4,210,000	1.001
1957	5,428,000	4,296,000	4,300,000	0.999
1958	5,377,000	4,229,000	4,246,000	0.996
1959	5,427,000	4,256,000	4,286,000	0.993
1960	5,481,000	4,258,000	4,257,850	1.000
1961	5,441,000	4,247,000	4,268,326	0.995
1962	5,420,000	4,144,000	4,167,362	0.994
1963	5,371,000	4,068,000	4,098,020	0.993
1964	5,307,000	4,000,000	4,027,490	0.993
1965	5,045,000	3,736,000	3,760,358	0.994
1966	4,875,000	3,586,000	3,606,274	0.994
1967	4,804,000	3,509,000	3,520,959	0.997
1968	4,835,000	3,498,000	3,501,564	0.999
1969	4,930,000	3,605,000	3,600,206	1.001
1970	5,091,000	3,750,000	3,737,800	1.003
1971	4,920,000	3,583,000	3,563,548	1.005
1972	4,652,000	3,298,000	3,266,235	1.010
1973	4,498,000	3,179,000	3,146,125	1.010
1974	4,521,000	3,207,000	3,170,631	1.011
1975	4,490,000	3,194,000	3,153,556	1.013
1976	4,504,000	3,214,000	3,176,476	1.012
1977	4,648,000	3,364,000	3,332,159	1.010
1978	4,639,000	3,361,000	3,338,300	1.007
1979	4,802,000	3,523,000	3,499,795	1.007
1980	4,922,000	3,643,000	3,617,981	1.007
1981	4,909,000	3,657,000	3,635,515	1.006
1982	4,982,000	3,707,000	3,685,457	1.006
1983	4,912,000	3,659,000	3,642,821	1.004
1984	4,921,000	3,685,000	3,673,568	1.003
1985	4,994,000	3,776,000	3,765,064	1.003
1986	4,969,000	3,772,000	3,760,695	1.003
1987	5,006,000	3,829,000	3,813,216	1.004
1988	5,089,000	3,929,000	3,913,793	1.004
1989	5,197,000	4,091,000	4,045,693	1.011
1990	5,279,000	4,208,000	4,162,917	1.011
1991	5,159,000	4,154,000	4,115,342	1.009
1992	5,079,000	4,105,000	4,069,428	1.009
1993	4,961,000	4,038,000	4,004,523	1.008
1994	4,864,000	3,988,000	3,956,925	1.008
1995	4,756,000	3,933,000	3,903,012	1.008
1996	4,684,000	3,921,000	3,894,874	1.007
1997	4,609,000	3,907,000	3,884,329	1.006
1998	4,589,000	3,966,000	3,945,192	1.005
1999	4,522,000	3,986,000	3,963,465	1.006
2000	4,550,000	4,084,000	4,063,823	1.005
2001	4,429,000	4,049,000	4,031,531	1.004
2002	4,376,000	4,041,000	4,027,376	1.003
2003	4,418,000	4,107,000	4,096,092	1.003
2004	4,429,000	4,130,000	4,118,907	1.003
2005	4,440,000	4,157,000	4,145,619	1.003
2006	4,551,000	4,282,000	4,273,225	1.002
2007	4,590,000	4,330,000	4,324,008	1.001
2008	4,510,000	4,262,000	4,255,156	1.002
2009	4,383,000	4,144,000	4,137,836	1.001
2010	4,238,000	4,013,000	4,007,105	1.001
2011	4,177,000	3,967,000	3,961,220	1.001
2012	4,166,000	3,966,000	3,960,796	1.001
2013	4,125,000	3,946,000	3,940,764	1.001
2014	4,169,000	4,006,000	3,998,175	1.002
2015	4,134,000	3,994,000	3,988,733	1.001
2016	4,077,000	3,961,000	3,956,112	1.001
2017	3,965,000	3,874,000	3,864,754	1.002
2018	3,874,000	3,802,000	3,801,534	1.000

*Notes*: Census Numident calculations from vintage 2020Q3. All Census Numident results were approved for release by the US Census Bureau, authorization number CBDRB-FY21-ERD002-016. These CDC NVSS birth counts for births occurring in the United States for 1979 to the present are from the published Natality Public Use File Documentation. For 1971–1978, counts were obtained from the Vital Statistics of the United States Volume I, Natality annual reports. For all years prior to 1971, published counts were obtained from Table 1-1, Live Births, Birth Rates, and Fertility Rates, by Race: United States, 1909–2000.

**Table 2:** Births by state of occurrence, 2009–2018

	(1)	(2)	(3)
	Census Numident	CDC NVSS	Census Numident/CDC NVSS
United States	39,670,000	39,617,029	1.001
Alabama	579,000	579,111	1.000
Alaska	110,000	110,137	0.999
Arizona	872,000	865,903	1.007
Arkansas	375,000	372,605	1.006
California	4,957,000	4,957,577	1.000
Colorado	663,000	661,700	1.002
Connecticut	375,000	375,377	0.999
Delaware	115,000	114,522	1.004
District of Columbia	142,000	141,982	1.000
Florida	2,195,000	2,194,683	1.000
Georgia	1,328,000	1,326,947	1.001
Hawaii	184,000	184,389	0.998
Idaho	222,000	222,496	0.998
Illinois	1,543,000	1,542,842	1.000
Indiana	846,000	844,201	1.002
Iowa	389,000	387,447	1.004
Kansas	402,000	401,016	1.002
Kentucky	534,000	533,697	1.001
Louisiana	630,000	629,019	1.002
Maine	125,000	125,385	0.997
Maryland	739,000	702,395	1.052
Massachusetts	724,000	724,909	0.999
Michigan	1,129,000	1,125,183	1.003
Minnesota	684,000	684,378	0.999
Mississippi	381,000	380,910	1.000
Missouri	766,000	763,846	1.003
Montana	121,000	121,144	0.999
Nebraska	266,000	265,689	1.001
Nevada	356,000	355,633	1.001
NewHampshire	126,000	125,848	1.001
NewJersey	1,017,000	1,016,518	1.000
NewMexico	250,000	249,846	1.001
NewYork	2,384,000	2,388,942	0.998
NorthCarolina	1,224,000	1,225,574	0.999
NorthDakota	119,000	118,313	1.006
Ohio	1,395,000	1,395,043	1.000
Oklahoma	515,000	513,740	1.002
Oregon	455,000	455,266	0.999
Pennsylvania	1,403,000	1,405,207	0.998
RhodeIsland	116,000	115,561	1.004
SouthCarolina	547,000	546,248	1.001
SouthDakota	127,000	127,187	0.999
Tennessee	863,000	861,011	1.002
Texas	3,978,000	3,975,120	1.001
Utah	520,000	518,508	1.003
Vermont	56,500	56,572	0.999
Virginia	1,010,000	1,011,171	0.999
Washington	879,000	877,234	1.002
WestVirginia	206,000	204,434	1.008
Wisconsin	663,000	666,818	0.994
Wyoming	68,000	67,745	1.004
Unknown	40	-	
USTerritories	412,000	405,424	1.016

*Notes*: Census Numident calculations from vintage 2020Q3. All Census Numident results were approved for release by the US Census Bureau, authorization number CBDRB-FY21-ERD002-016. The CDC NVSS birth counts were obtained from the published Natality Public Use File Documentation.

**Table 3:** Yearly births linked to parents in the Census Household Composition Key (CHCK), 1997–2018

				For births with parent link		
	(1)	(2)	(3)	(4)	(5)	(6)
Birth year	Total births	Linked to a parent	% of total births with parent link	% linked to mother only	% linked to father only	% linked to mother and father
1997	4,384,000	4,053,000	92.45%	32.59%	4.59%	62.82%
1998	4,406,000	4,122,000	93.55%	31.05%	4.00%	64.94%
1999	4,440,000	4,218,000	95.00%	18.07%	2.61%	79.33%
2000	4,516,000	4,284,000	94.86%	16.36%	2.68%	80.95%
2001	4,403,000	4,196,000	95.30%	15.99%	2.79%	81.22%
2002	4,309,000	4,114,000	95.47%	15.65%	2.80%	81.53%
2003	4,405,000	4,203,000	95.41%	15.63%	2.81%	81.56%
2004	4,416,000	4,210,000	95.34%	15.39%	2.83%	81.76%
2005	4,429,000	4,215,000	95.17%	15.44%	2.89%	81.66%
2006	4,540,000	4,313,000	95.00%	15.81%	2.92%	81.27%
2007	4,579,000	4,341,000	94.80%	15.71%	2.88%	81.41%
2008	4,499,000	4,272,000	94.95%	15.45%	2.81%	81.74%
2009	4,371,000	4,158,000	95.13%	15.08%	2.67%	82.23%
2010	4,226,000	4,005,000	94.77%	14.76%	2.70%	82.55%
2011	4,166,000	3,964,000	95.15%	14.73%	2.65%	82.62%
2012	4,154,000	3,962,000	95.38%	14.71%	2.62%	82.64%
2013	4,113,000	3,929,000	95.53%	13.97%	2.55%	83.51%
2014	4,157,000	3,918,000	94.25%	13.14%	2.58%	84.28%
2015	4,122,000	3,825,000	92.79%	15.27%	3.03%	81.67%
2016	4,066,000	3,688,000	90.70%	16.73%	3.42%	79.88%
2017	3,954,000	3,464,000	87.61%	17.87%	3.75%	78.38%
2018	3,857,000	3,090,000	80.11%	20.06%	4.95%	74.98%

*Notes*: CHCK calculations from 2016–2019 CHCK files. All results were approved for release by the U.S. Census Bureau, authorization number CBDRB-FY21-ERD002-016.

**Table 4:** Demographic characteristics by dataset for birth years 1997–2018

	(1)	(2)	(3)
	Linked to Parent in CHCK	Census Numident	Weighted 2019 ACS
**Sex**			
Female	48.86%	48.85%	48.77%
Male	51.14%	51.15%	51.23%
**Race/Ethnicity**			
Black, Non-Hispanic	13.16%	13.03%	13.54%
White, Non-Hispanic	54.61%	53.65%	50.79%
Asian, Non-Hispanic	4.19%	4.14%	5.04%
AIAN/NHPI, Non-Hispanic	1.00%	1.00%	0.76%
Hispanic	22.69%	23.89%	24.84%
Other/Multiple, Non-Hispanic	4.36%	4.30%	5.04%
**Birthplace**			
Born in US	95.16%	93.68%	93.65%
Born in US territory	1.04%	1.18%	0.36%
Born Abroad	3.81%	5.14%	5.99%

*Notes*: CHCK calculations in Column 1 are from 2016–2019 CHCK files, and the Census Numident calculations are from vintage 2020Q3. Sex and Birthplace for the CHCK and Census Numident were obtained from the Census Numident with sex. Race was obtained for only those cases that linked to the 2010 or 2000 Decennial Census data, with 2010 race information being used if found in both. All Census Numident and CHCK results were approved for release by the US Census Bureau, authorization number CBDRB-FY21-ERD002-016. The weighted 2019 1-year ACS estimates (column 3) were calculated using data from IPUMS (Ruggles et al. 2020).

**Table 5:** CHCK linkage for American Community Survey (ACS) respondents under age 19 and born after 1996

				Panel A: Linked to mother in CHCK					Panel B: Linked to father in CHCK				
ACS survey year	(1)	(2)	(3)	(4)	(5)	(6)	(7)	(8)	(9)	(10)	(11)	(12)	(13)
	Total	Total with PIK assigned	% with PIK assigned	Linked to mother	*%* of total with PIK	Resides with linked mother	% of total with PIK residing with linked mother	% of those linked to mother residing with mother	Linked to father	*%* of total with PIK	Resides with linked father	% of total with PIK residing with linked father	% of those linked to father residing with father
2005	476,000	426,000	89.50%	406,000	95.31%	366,000	85.92%	90.15%	350,000	82.16%	290,000	68.08%	82.86%
2006	544,000	486,000	89.34%	463,000	95.27%	416,000	85.60%	89.85%	401,000	82.51%	328,000	67.49%	81.80%
2007	590,000	524,000	88.81%	501,000	95.61%	449,000	85.69%	89.62%	436,000	83.21%	355,000	67.75%	81.42%
2008	642,000	569,000	88.63%	542,000	95.25%	486,000	85.41%	89.67%	473,000	83.13%	382,000	67.14%	80.76%
2009	689,000	591,000	85.78%	564,000	95.43%	499,000	84.43%	88.48%	493,000	83.42%	392,000	66.33%	79.51%
2010	741,000	686,000	92.58%	649,000	94.61%	583,000	84.99%	89.83%	565,000	82.36%	446,000	65.01%	78.94%
2011	859,000	788,000	91.73%	748,000	94.92%	666,000	84.52%	89.04%	649,000	82.36%	504,000	63.96%	77.66%
2012	1,024,000	945,000	92.29%	902,000	95.45%	801,000	84.76%	88.80%	785,000	83.07%	607,000	64.23%	77.32%
2013	1,019,000	941,000	92.35%	900,000	95.64%	795,000	84.48%	88.33%	790,000	83.95%	609,000	64.72%	77.09%
2014	1,119,000	1,033,000	92.31%	987,000	95.55%	860,000	83.25%	87.13%	868,000	84.03%	658,000	63.70%	75.81%
2015	1,170,000	1,073,000	91.71%	1,023,000	95.34%	880,000	82.01%	86.02%	903,000	84.16%	678,000	63.19%	75.08%
2016	1,157,000	1,042,000	90.06%	995,000	95.49%	844,000	81.00%	84.82%	886,000	85.03%	661,000	63.44%	74.60%
2017	1,109,000	998,000	89.99%	954,000	95.59%	808,000	80.96%	84.70%	859,000	86.07%	639,000	64.03%	74.39%
2018	1,091,000	995,000	91.20%	949,000	95.38%	807,000	81.11%	85.04%	860,000	86.43%	639,000	64.22%	74.30%
2019	1,022,000	937,000	91.68%	869,000	92.74%	736,000	78.55%	84.70%	790,000	84.31%	588,000	62.75%	74.43%

*Notes*: CHCK calculations from 2016–2019 CHCK files and 2005 through 2019 1-year ACS data were used. All results were approved for release by the U.S. Census Bureau, authorization number CBDRB-FY21-ERD002-016.

**Table 6:** Comparison of ACS fertility question and CHCK-assigned births for ACS respondents

		ACS fertility question birth			CHCK birth			ACS fertility and CHCK birth		
	(1)	(2)	(3)	(4)	(5)	(6)	(7)	(8)	(9)	(10)
ACS Year	Total respondents in fertilty question universe	Total respondents indicating birth in last year	Percent of eligible women reporting births in last year	Percent of women reporting births in last year living with a child under age 1	Total respondents with birth assigned in CHCK in universe	Percent of eligible women with CHCK birth in last year	Percent of women with CHCK birth in last year living with CHCK indicated child	Total respondents indicating birth in last year *and* birth in CHCK	Percent of eligible women assigned birth in fertilty question and in CHCK	Percent of women indicating birth in ACS last year assigned birth in last year in CHCK
2005	1,076,000	58,000	5.39%	81.03%	46,000	4.28%	85.11%	41,500	3.86%	71.55%
2006	1,126,000	60,000	5.33%	80.83%	47,500	4.22%	85.42%	42,500	3.77%	70.83%
2007	1,092,000	58,500	5.36%	80.34%	46,000	4.21%	85.11%	41,500	3.80%	70.94%
2008	1,067,000	61,000	5.72%	78.69%	45,500	4.26%	85.87%	41,000	3.84%	67.21%
2009	1,050,000	58,000	5.52%	79.31%	42,500	4.05%	54.65%	38,500	3.67%	66.38%
2010	1,038,000	56,000	5.39%	78.57%	43,000	4.14%	88.51%	38,500	3.71%	68.75%
2011	1,143,000	60,500	5.29%	76.03%	45,000	3.94%	82.42%	40,500	3.54%	66.94%
2012	1,257,000	66,500	5.29%	76.69%	50,500	4.02%	87.25%	45,000	3.58%	67.67%
2013	1,177,000	59,500	5.06%	78.99%	47,000	3.99%	85.42%	41,000	3.48%	68.91%
2014	1,208,000	61,500	5.09%	79.67%	47,000	3.89%	77.32%	39,000	3.23%	63.41%
2015	1,186,000	60,000	5.06%	79.17%	47,000	3.96%	85.42%	41,500	3.50%	69.17%
2016	1,137,000	57,500	5.06%	80.00%	44,500	3.91%	87.78%	40,000	3.52%	69.57%
2017	1,094,000	56,500	5.16%	76.11%	41,500	3.79%	89.16%	37,500	3.43%	66.37%
2018	1.083,000	55,500	5.12%	74.77%	38,500	3.55%	91.03%	35,500	3.28%	63.96%

*Notes*: CHCK calculations from 2016–2019 CHCK files and 2005 through 2019 1-year ACS data were used. All results were approved for release by the U.S. Census Bureau, authorization number CBDRB-FY21-ERD002-016. The very low percentage of women with a birth in the CHCK and living with that indicated child in the ACS in 2009 (Column 7) is the result of extrememly low rates of PIK assignment of infants in the 2009 ACS.

## Footnotes

3 Adoptive parents are encouraged to apply for new SSNs for adoptive children for tax purposes: https://www.ssa.gov/pubs/EN-05-10023.pdf.

4 The Census Bureau does not have access to the SSA KIDLINK file.

5 CHCK are generally released in February of the following year, so the 2019 CHCK was made available to researchers in February 2020.

6 All analyses were performed using SAS version 9.9.

7 All counts from the Census Numident are rounded according to the Census Bureau’s Disclosure Review Board’s rounding rules.

8 We compiled CDC NVSS birth counts for births occurring in the U.S. by capturing yearly counts for 1979 to the present from the published Natality Public Use File Documentation downloaded from NBER (https://www.nber.org/research/data/vital-statistics-natality-birth-data) or the CDC NVSS website (https://www.cdc.gov/nchs/data_access/vitalstatsonline.htm). For 1971–1978, counts were obtained from the Vital Statistics of the United States Volume I, Natality annual reports (https://www.cdc.gov/nchs/products/vsus.htm). For all years prior to 1971, published counts were obtained from Table 1-1. Live Births, Birth Rates, and Fertility Rates, by Race: United States, 1909–2000 (https://www.cdc.gov/nchs/data/statab/t001x01.pdf).

9 The Census Numident includes births before 1910.

10 Births by state of occurrence for each year were obtained from the tables published in Natality Public Use File Documentation, downloaded from NBER (https://www.nber.org/research/data/vital-statistics-natality-birth-data) or the CDC NVSS website (https://www.cdc.gov/nchs/data_access/vitalstatsonline.htm).

11 In addition to state, the Census Numident includes a more detailed place of birth, which is often a city, and researchers have created a GNIS code crosswalk for the place of birth information in the Census Numident. Specific U.S. territory of birth is also available.

12 A small number of children link to new parents in subsequent CHCK files, as a result of changes in parents names in the SSA source data. Investigation of the data by the authors showed there were substantial updates to the source data starting in 2018, and the authors suggest using the most recent links of parents to children as they are the most accurate.

13 The total births in Table 3 are not identical to the total births in the Census Numident reported in Table 1; this is due to the variation in vintages of the Census Numident used to create each CHCK file. We use the 2020Q3 vintage of the Census Numident in Table 1, which is also more recent than the 2016–2019 CHCK files.

14 Legal parents of the same sex can have both names on an SSN application (https://www.ssa.gov/people/same-sexcouples/), but the 2016–2019 CHCK files limit the mother and father links by sex.

15 In a small number of cases, parental information comes from the SSA’s application for a Social Security Card (Form SS-5).

16 The ACS fertility question states, “Has this person given birth to any children in the past 12 months?” It also includes the following in the instructions: “Mark the ‘Yes’ box if the person has given birth to at least one child born alive in the past 12 months, even if the child died or no longer lives with the mother. Do not consider miscarriages, or stillborn children, or any adopted, foster, or stepchildren.” We calculate the previous 12 months based on the date the ACS questionnaire was completed. Future work will investigate the 10% of women with reported births who do not coreside with an infant in the ACS.

17 FSRDC locations and contact information: https://www.census.gov/about/adrm/fsrdc/locations.html

18 The Census Bureau’s FSRDC research proposal guidelines and processes: https://www.census.gov/about/adrm/ced/apply-for-access.html.
